# Changing Patterns of HCV Genotype Distribution in a Migration-Affected Region of Turkey: A Retrospective Hospital-Based Study (2014–2021)

**DOI:** 10.3390/v18050529

**Published:** 2026-04-30

**Authors:** Yasemin Ay Altintop, Esma Saatçi

**Affiliations:** Department of Medical Microbiology, Kayseri City Training and Research Hospital, University of Health Sciences, 38080 Kayseri, Türkiye; dresmasaatci@hotmail.com

**Keywords:** hepatitis C, genotype distribution, migration, refugee populations, molecular epidemiology, Central Anatolia, viral evolution, public health, HCV RNA

## Abstract

Hepatitis C virus (HCV) infection is still a major worldwide health concern. It is distinguished by a high degree of genetic variation that affects the course of the illness and the effectiveness of treatment. The epidemiological profile of HCV is prone to rapid change in areas where there is significant human migration, like Turkey. The purpose of this study was to evaluate the impact of long-term migration on local viral diversity by analyzing the distribution and temporal trends of HCV genotypes among Turkish citizens and asylum seekers in Kayseri, Turkey, over an eight-year period. From January 2014 to December 2021. 1173 HCV RNA-positive patients at Kayseri City Training and Research Hospital were the subject of a retrospective analysis. Genotypes were determined using the Abbott RealTime HCV Genotype II assay and Montania 4896 assay (Anatolia Geneworks, Türkiye). The most prevalent genotypes were Genotype 1b (48.3%, 95% CI: 45.5–51.2%), Genotype 4 (25.0%, 95% CI: 22.5–27.5%), and Genotype 1a (10.3%, 95% CI: 8.6–12.1%). Turkish patients exhibited the highest prevalence of Genotype 1b (98.2%), while asylum seekers demonstrated greater relative burdens of Genotype 4 (8.5% of total GT4) and Genotype 5 (83.3% of total GT5). Genotype 3a emerged in 2018, with a predominance in males (73.9%). The Cochran–Armitage trend test revealed statistically significant increasing trends for Genotype 3 (Z = 3.572, *p* = 0.0004) and Genotype 3a (Z = 2.600, *p* = 0.009). This eight-year retrospective study demonstrates that the HCV genotype distribution in Kayseri has undergone significant changes in the context of migration and demographic shifts. The statistically significant increasing trends of Genotypes 3 and 3a, particularly among younger male populations, suggest evolving transmission dynamics. These findings underscore the necessity of demographically targeted and culturally appropriate screening and treatment strategies for both resident and migrant populations to achieve HCV elimination goals.

## 1. Introduction

Globally, an estimated 58 million individuals are afflicted by chronic hepatitis C virus (HCV) infection, thereby constituting a significant global health challenge [1]. The World Health Organization (WHO) attributes the annual mortality of 290,000 deaths caused by chronic hepatitis C to severe outcomes such as cirrhosis and hepatocellular carcinoma (HCC) [2]. HCV, a member of the Flaviviridae family, is known for its considerable genetic diversity.

It includes seven main genotypes (1–7) and over eighty subtypes, with genomic nucleotide divergence exceeding 30% [3]. This genetic variation significantly affects how the virus behaves, how well treatments work, and how the disease spreads.

The distribution of HCV genotypes varies greatly worldwide, reflecting the influence of different demographic and geographic factors. In industrialized nations, genotypes 1a, 1b, 2a, and 3a are often observed; however, genotypes 1 and 2 are more common in West Africa. Conversely, genotype 3 is prevalent in South Asia, genotype 4 in Central Africa and the Middle East, genotype 5 in Southern Africa, and genotype 6 in Southeast Asia [4]. In North America, Genotype 1a is the most prevalent genotype, accounting for approximately 60–70% of all HCV infections, followed by Genotype 1b. In South America, the distribution varies by region; Genotype 1 predominates in Brazil, Argentina, and Chile, while Genotype 3 has shown an increasing prevalence, particularly in association with intravenous drug use [5]. In Western Europe, Genotype 1b has historically been the most common genotype, largely associated with the era of transfusion-related transmission. However, in recent years, Genotype 3 has shown a marked increase in the United Kingdom, Scandinavian countries, and Australia, particularly among younger populations and people who inject drugs. In Eastern Europe, Genotype 1b remains predominant, while in Southern Europe (Italy, Spain), both Genotype 1b and Genotype 2 exhibit high prevalence. Furthermore, due to migration patterns within Europe, genotypes originating from the Middle East and Africa (Genotypes 4 and 5) are being increasingly detected in European countries [6].

The evolving nature of HCV epidemiology, along with the impact of human migration on viral spread, is underscored by the recent identification of Genotype 7 in an African immigrant residing in Canada [7]. Although current direct-acting antivirals (DAAs) are largely pangenotypic, with regimens such as sofosbuvir/velpatasvir and glecaprevir/pibrentasvir demonstrating high efficacy across all major genotypes, accurate genotype identification remains essential for epidemiological surveillance, understanding transmission dynamics, predicting potential treatment challenges in specific subtypes, and guiding public health strategies [8].

Historically, Genotype 1, and specifically Subtype 1b, has been the most common HCV genotype in Turkey [9]. In contrast, recent demographic changes, primarily due to the arrival of migrants and asylum seekers from conflict-affected regions, particularly Syria, have created new epidemiological challenges. Turkey currently hosts the largest refugee population in the world, with over 3.6 million registered refugees, the majority of whom are of Syrian origin [10]. Throughout this manuscript, the term ‘asylum seekers’ is used to refer to non-Turkish nationals, as migration status was determined based on nationality records in the hospital information system. Given that migrant populations often carry HCV genotypes common in their home countries, this significant population movement could potentially alter the local distribution of HCV genotypes [11].

The World Health Organization (WHO) aims to eliminate hepatitis C virus (HCV) as a public health threat by 2030, targeting a 90% reduction in new infections and a 65% reduction in mortality relative to a 2015 baseline [12]. Achieving these ambitious goals requires a thorough understanding of local genotype distributions and their temporal changes, particularly in regions experiencing significant demographic shifts due to migration.

The purpose of this study was to evaluate the impact of long-term migration on local viral diversity by analyzing the distribution and temporal trends of HCV genotypes among Turkish citizens and asylum seekers in Kayseri, Turkey, over an eight-year period (2014–2021).

## 2. Methods

### 2.1. Population and Study Design

Kayseri is a province located in the Central Anatolia region of Turkey with a population of approximately 1.4 million, serving as a significant industrial and healthcare center. Due to its proximity to the Syrian border, the city has been directly affected by migration movements. Kayseri Training and Research Hospital is a publicly funded, tertiary-level healthcare institution affiliated with the Health Sciences University (SBÜ). It is a postgraduate medical training and research hospital and serves as the largest referral center in the region, accepting patients from surrounding provinces.

Under Turkey’s universal health insurance system, both Turkish citizens and asylum seekers under temporary protection status have access to public healthcare services free of charge. Patients can access the hospital either through referral from primary healthcare facilities or through direct admission.

This study was designed and conducted by laboratory specialists working in the Medical Microbiology Laboratory. Patients with HCV RNA who were referred to the Medical Microbiology Laboratory at Kayseri Training and Research Hospital between January 2014 and December 2021 were the subjects of this retrospective cohort analysis. Both Turkish citizens and asylum seekers living in Kayseri made up the study population. The local ethics committee granted ethical approval for this retrospective analysis (Approval no: 482, Date: 7 April 2022). Throughout the investigation, patient confidentiality was protected by anonymizing all information gathered.

### 2.2. HCV RNA Identification and Genotyping

The COBAS^®^ AmpliPrep/COBAS^®^ TaqMan^®^ system (Roche Molecular Systems, Inc., Branchburg, NJ, USA) was used to detect HCV RNA in accordance with the manufacturer’s instructions. Two CE-IVD certified genotyping assays were used during the study period: the Abbott RealTime HCV Genotype II assay (Abbott Molecular Inc., Des Plaines, IL, USA) and the Montania 4896 assay (Anatolia Geneworks, İstanbul, Türkiye). The change in genotyping platform occurred due to routine laboratory procurement transitions during the eight-year study period. Each sample was genotyped using the assay that was routinely in use at the time of testing; not all samples were tested with both methods. Both assays enable the detection of HCV genotypes 1, 2, 3, 4, 5, and 6 as well as subtypes 1a and 1b. To avoid data duplication, only the first positive result for patients with multiple HCV RNA testing was included in the study.

### 2.3. Data Collection

**Figure 1:**
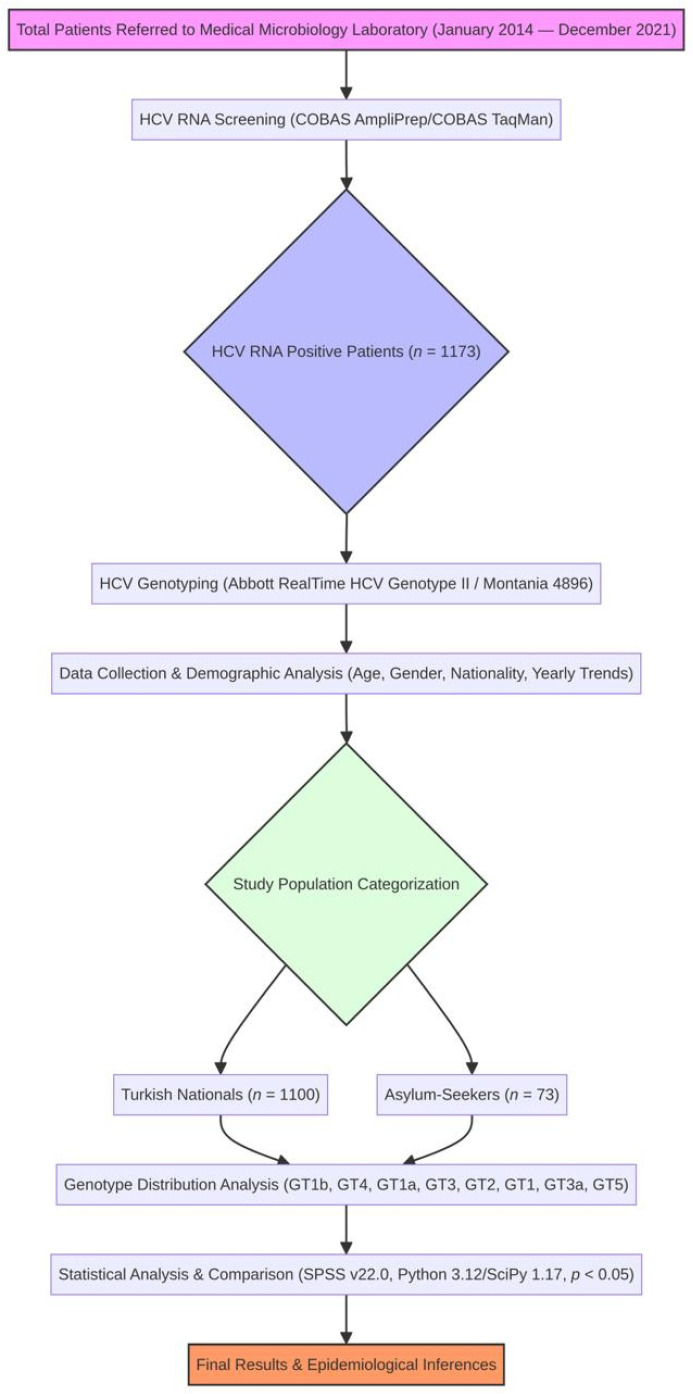
Flow chart of the study.

Age, gender, and nationality (classified as Turkish citizens or asylum seekers) were among the clinical and demographic information that was carefully taken out of the electronic patient records (Figure 1). Migration status was determined based on nationality records available in the hospital electronic information system. As this study was conducted by laboratory specialists, detailed clinical and demographic migration data such as length of stay, registration status, or migration history were not available, as these are not routinely documented in laboratory records. Prior to analysis, all data underwent de-identification. The study sample was subsequently stratified into two principal cohorts: Turkish citizens (*n* = 1100) and asylum seekers (*n* = 73). The majority of asylum seekers originated from Syria and neighbouring conflict-affected regions.

### 2.4. Statistical Analysis

Statistical analyses were performed using SPSS software (version 22.0; IBM SPSS Statistics, Armonk, NY, USA) and Python (version 3.12; Python Software Foundation). Descriptive statistics were utilized to compile the genotypic and demographic characteristics of the study population. Continuous variables were reported as medians (interquartile range), while categorical variables were summarized using frequencies and percentages. The chi-square test was implemented to evaluate genotype distributions concerning categorical variables. Non-parametric tests (Mann–Whitney U test) were applied for continuous variables. Statistical significance was defined as a two-sided *p*-value of less than 0.05. Additionally, 95% confidence intervals (CIs) were calculated for major genotype proportions. The Cochran–Armitage trend test was applied to assess the statistical significance of year-on-year changes in genotype distribution. Cramér’s V was calculated as a measure of effect size for categorical associations.

## 3. Results

A total of 1173 HCV RNA-positive individuals were included in the analysis during the eight-year research period, which ran from January 2014 to December 2021. Table 1 displays the demographic and virological characteristics of the study population stratified by nationality.

### 3.1. Distribution of Genotypes

**Figure 2:**
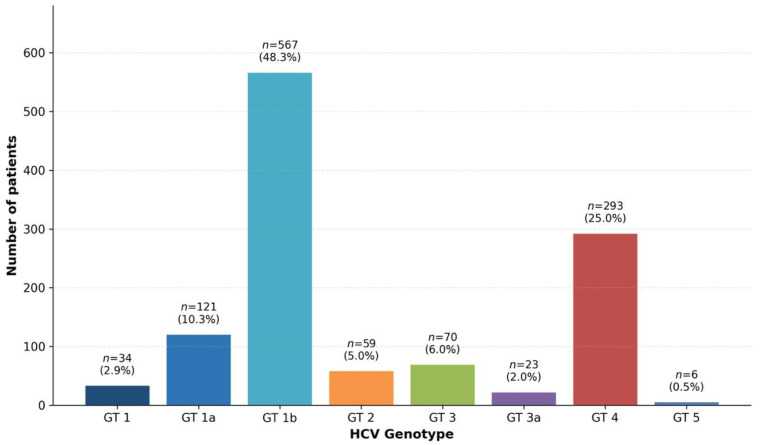
Overall HCV genotype distribution among 1173 HCV RNA-positive patients in Kayseri, Turkey (2014–2021). Bars represent the number of patients per genotype, with percentages shown above each bar. Genotype 1b was the most prevalent (48.3%), followed by Genotype 4 (25.0%) and Subtype 1a (10.3%).

The overall distribution of HCV genotypes among the 1173 HCV RNA-positive individuals is illustrated in Figure 2. Genotype 1b was the most prevalent genotype, identified in 567 patients (48.3%, 95% CI: 45.5–51.2%), followed by Genotype 4 in 293 patients (25.0%, 95% CI: 22.5–27.5%), and Genotype 1a in 121 patients (10.3%, 95% CI: 8.6–12.1%). Less frequently observed genotypes included Genotype 3 (*n* = 70; 6.0%, 95% CI: 4.6–7.3%), Genotype 2 (*n* = 59; 5.0%, 95% CI: 3.8–6.3%), Genotype 1 (*n* = 34; 2.9%, 95% CI: 1.9–3.9%), Genotype 3a (*n* = 23; 2.0%, 95% CI: 1.2–2.8%), and Genotype 5 (*n* = 6; 0.5%, 95% CI: 0.1–0.9%). Collectively, subtypes of Genotype 1 (including Genotype 1, 1a, and 1b) accounted for 61.5% of all cases, making Genotype 1 and its subtypes the dominant viral lineage in the study population.

### 3.2. Distribution of Gender

**Figure 3:**
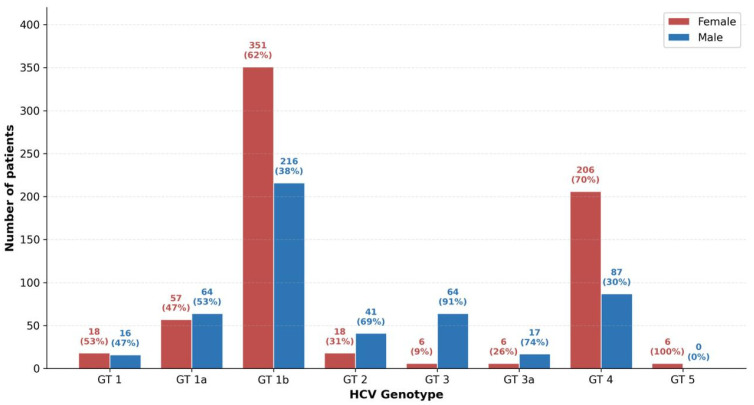
Gender distribution of HCV genotypes among 1173 patients in Kayseri, Turkey (2014–2021). Red bars represent female patients (*n* = 668) and blue bars represent male patients (*n* = 505). Numbers and percentages above each bar indicate within-genotype gender proportions. Genotypes 1b and 4 showed female predominance, while Genotypes 3 and 3a exhibited a marked male predominance.

Of the 1173 patients included in the study, 668 (56.9%) were female and 505 (43.1%) were male, indicating a slight female predominance in the overall cohort. The distribution of genotypes by gender is illustrated in Figure 3. Notable gender-related differences were observed across genotypes. Genotype 1b was more common in females, with 351 of 567 cases (61.9%) occurring in women. Similarly, Genotype 4 demonstrated a pronounced female predominance, with 206 of 293 cases (70.3%) in women. In contrast, Genotypes 3 and 3a exhibited a significant male predominance: 64 of 70 Genotype 3 cases (91.4%) and 17 of 23 Genotype 3a cases (73.9%) occurred in men. Genotype 2 was also more frequently observed in males (41 of 59 cases, 69.5%). Gender distributions were comparatively balanced in Subtype 1a (males 52.9%, females 47.1%) and Genotype 1 (males 47.1%, females 52.9%). Notably, all six Genotype 5 cases (100%) were identified in female patients. The overall association between gender and genotype distribution was statistically significant (χ^2^ = 129.12, *p* < 0.001, Cramér’s V = 0.332, indicating a medium effect size).

### 3.3. Distribution Based on Nationality

The study population consisted of 1100 Turkish citizens (93.8%) and 73 asylum seekers (6.2%). Notably, this proportion of asylum seekers is consistent with the actual ratio of the migrant population to the total population in Kayseri province (approximately 6%), indicating that our study sample reflects the true demographic composition of the region. The genotype distribution differed considerably between the two groups. Among Turkish patients, Genotype 1b was the dominant genotype (557 of 1100 cases, 50.6%), followed by Genotype 4 (268 cases, 24.4%) and Subtype 1a (104 cases, 9.5%). Looking at the proportions within each genotype, Turkish patients accounted for 98.2% of all Genotype 1b cases, 91.5% of all Genotype 4 cases, and 86.0% of all Subtype 1a cases.

In contrast, the asylum seeker cohort demonstrated a markedly different genotype profile. Genotype 4 was the most prevalent (25 of 73 cases, 34.2%), followed by Subtype 1a (17 cases, 23.3%), Subtype 1b (10 cases, 13.7%), and Genotype 3 (9 cases, 12.3%). Examining the relative burden across the total study population, asylum seekers accounted for disproportionately high proportions of Genotype 5 (83.3% of all Genotype 5 cases), Genotype 3a (21.7% of all Genotype 3a cases), Subtype 1a (14.0% of all Subtype 1a cases), and Genotype 3 (12.9% of all Genotype 3 cases). Notably, five of the six Genotype 5 cases in the study were identified in asylum seekers. The overall association between nationality and genotype distribution was statistically significant (χ^2^ = 113.30, *p* < 0.001, Cramér’s V = 0.311, indicating a medium effect size). These findings suggest that migration may have contributed to the introduction and enrichment of specific HCV genotypes within the local epidemiological landscape.

### 3.4. Distribution of Ages

**Figure 4:**
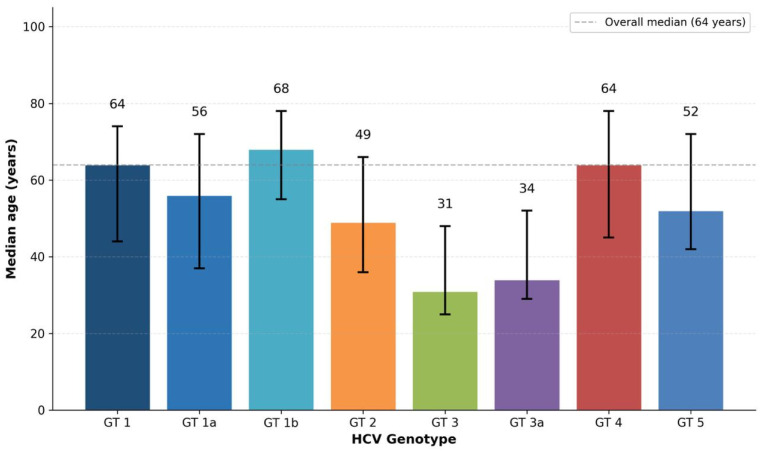
Median age distribution by HCV genotype with interquartile range (IQR) error bars. The horizontal dashed line represents the overall median age (64 years). Genotypes 3 and 3a showed significantly lower median ages (31 and 34 years, respectively), while Genotype 1b exhibited the highest median age (68 years). The age difference between Genotype 3/3a and Genotype 1b was statistically significant (*p* < 0.001).

The overall median age of the study population was 64 years (range: 14–92 years), reflecting a predominantly older demographic. Age distribution by genotype is illustrated in Figure 4. Marked age-related differences were observed across genotypes. Patients infected with Genotype 3 and Genotype 3a were considerably younger, with median ages of 31 years (range: 19–72) and 34 years (range: 24–76), respectively. This younger age profile contrasts sharply with the older median ages observed in other genotypes: Genotype 1b at 68 years (range: 19–91), Genotype 1 at 64 years (range: 24–83), Genotype 4 at 64 years (range: 14–92), and Subtype 1a at 56 years (range: 18–90). Genotype 2 showed an intermediate median age of 49 years (range: 23–83), while Genotype 5 cases had a median age of 52 years (range: 39–85). The age difference between patients with Genotype 3/3a and those with Genotype 1b was statistically significant (*p* < 0.001). This age disparity suggests that Genotype 3 and 3a infections are associated with distinct, potentially more recent transmission dynamics compared to the older transmission patterns reflected by Genotype 1b, which was historically linked to iatrogenic exposures such as blood transfusions in earlier decades.

### 3.5. Temporal Trends in Genotype Distribution

**Figure 5:**
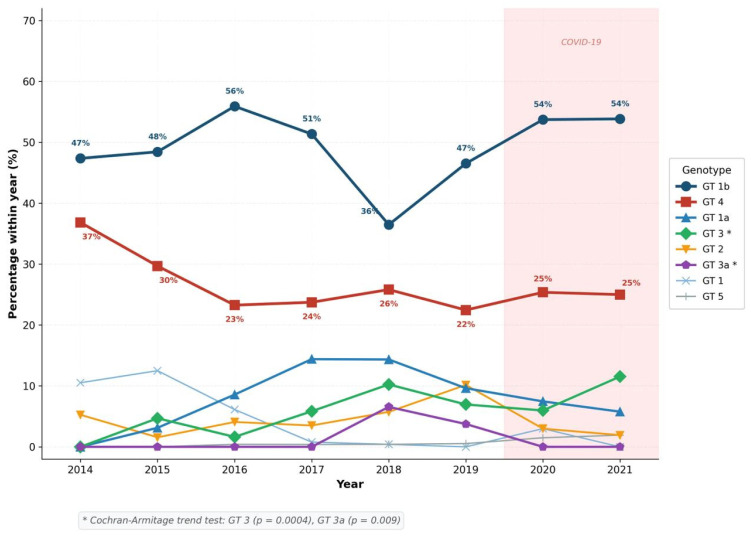
Temporal trends in HCV genotype distribution in Kayseri, Turkey (2014–2021), showing within-year percentage proportions. The shaded area indicates the COVID-19 pandemic period (2020–2021). Statistically significant increasing trends (Cochran–Armitage trend test, *p* < 0.05) were observed for Genotype 3 and Genotype 3a (marked with *). Genotype 1b remained the predominant genotype throughout the study period, while Genotype 4 maintained a stable presence. The marked decline in total case numbers after 2018 may reflect the combined effects of widespread direct-acting antiviral (DAA) adoption and reduced healthcare access during the COVID-19 pandemic.

The temporal trends in the within-year percentage distribution of HCV genotypes over the eight-year study period (2014–2021) are illustrated in Figure 5. The annual patient volume varied considerably: 2014 (*n* = 57), 2015 (*n* = 64), 2016 (*n* = 245), 2017 (*n* = 257), 2018 (*n* = 244), 2019 (*n* = 187), 2020 (*n* = 67), and 2021 (*n* = 52). Notably, the total number of HCV RNA-positive cases declined markedly from 2018 onwards, dropping from 244 cases in 2018 to 52 cases in 2021.

Several important temporal patterns emerged from the analysis. Subtype 1b demonstrated the highest within-year prevalence throughout most of the study period, peaking at 55.9% in 2016 before gradually declining to 36.5% in 2018, and then fluctuating between 46.5% and 53.8% in subsequent years. Although a declining trend was observed for Subtype 1b, this did not reach statistical significance (Z = −1.071, *p* = 0.284). In contrast, the Cochran–Armitage trend test revealed statistically significant increasing trends for Genotype 3 (Z = 3.572, *p* = 0.0004) and Genotype 3a (Z = 2.600, *p* = 0.009). Genotype 3 rose from 0% in 2014 to a peak of 11.5% in 2021, while Genotype 3a emerged abruptly in 2018 (6.6% of that year’s cases), persisted into 2019 (3.7%), and was absent in subsequent years.

Genotype 4 maintained a relatively stable within-year prevalence throughout the study period, ranging between 22.5% and 36.8%, indicating a sustained endemic reservoir within the study population. Subtype 1a showed a modest but non-significant increasing trend (Z = 1.338, *p* = 0.181), rising from 0% in 2014 to 14.4% in 2017 before declining. Genotype 2 showed variable prevalence, peaking at 10.2% in 2019. Genotype 5, though rare, appeared consistently from 2016 onwards in low numbers, predominantly among asylum seekers.

## 4. Discussion

In alignment with the World Health Organization’s (WHO) 2030 elimination targets, this study aims to contribute meaningfully to enhancing awareness and strengthening the ongoing fight against hepatitis C [12,13,14]. By documenting evolving patterns of HCV genotype distribution in a migration-affected region over an eight-year period, our findings provide a valuable epidemiological foundation for targeted screening strategies, surveillance systems, and public health interventions that can support the WHO’s goals of a 90% reduction in new infections and 65% reduction in mortality relative to the 2015 baseline. Importantly, the observed decline in HCV RNA-positive cases after 2018, and particularly during the COVID-19 pandemic period (2020–2021) [15,16], likely reflects the combined effects of several concurrent developments: (1) the widespread adoption of highly effective pangenotypic direct-acting antiviral (DAA) regimens [14], which have transformed HCV from a chronic incurable disease to a curable one; (2) enhanced infection prevention and control measures implemented during the pandemic, including universal mask use, strict hand hygiene protocols, reduced healthcare-associated procedures, and improved sterilization practices, which may have reduced nosocomial HCV transmission; (3) increased public awareness of bloodborne and infectious disease transmission routes during the pandemic era; and (4) expansion of national treatment programs and screening initiatives in line with WHO recommendations. These combined factors suggest that the pandemic period, despite its negative impact on healthcare access, may have inadvertently contributed to reductions in HCV transmission through heightened public health vigilance and expanded treatment uptake. Our findings thus underscore the importance of sustaining and expanding these preventive measures and treatment programs in the post-pandemic era to accelerate progress toward HCV elimination.

### 4.1. Summary of Main Findings

This eight-year retrospective study analyzed the distribution of HCV genotypes among 1173 HCV RNA-positive individuals in Kayseri, Turkey. The key findings include: (1) Genotype 1b remains the most prevalent genotype overall (48.3%) but is predominantly found in Turkish nationals (50.6%); (2) asylum seekers demonstrate a distinctly different genotype profile with higher proportions of Genotype 4 (34.2%), Subtype 1a (23.3%), and Genotype 5 (6.8%); (3) statistically significant increasing trends were observed for Genotype 3 and 3a, particularly among younger males; and (4) a notable decline in total HCV RNA-positive cases was observed from 2018 onwards.

### 4.2. Comparison with Existing Literature

The hepatitis C virus (HCV) exhibits significant genetic diversity and is transmitted through multiple well-characterized routes, including parenteral transmission via unsafe injection practices, inadequately screened blood transfusions, organ transplantation, nosocomial exposure, intravenous drug use (IDU), vertical transmission, sexual transmission, occupational needlestick injuries, and unregulated tattooing and piercing practices. The relative contribution of each route varies by region and period, influencing local genotype distribution [4,5,6].

Genotype 1b continues to be more common among Turkish citizens, consistent with historical data from Turkey and several European nations [9,17]. The predominance of Genotype 1b among Turkish females may reflect historical transmission patterns associated with blood transfusions and medical procedures that disproportionately affected women in earlier decades, particularly during childbirth and gynecological interventions. In contrast, among asylum seekers, a male predominance was observed, which may be attributable to differences in risk factor exposure during migration. However, as our study did not collect individual-level risk factor data, these interpretations remain speculative.

The emergence of Genotype 3a in 2018, primarily among men, highlights the changing nature of local HCV epidemiology. However, since Genotype 3a appeared suddenly in 2018 and was absent after 2019, it remains unclear whether this represents a true epidemiological shift or a transient cluster [17,18,19].

The continuous prevalence of Genotype 4 among asylum seekers may reflect the impact of population migration on local viral diversity, as Genotype 4 is endemic to the Middle East and neighbouring regions [11,20]. However, as migration status was determined solely based on nationality, these observations represent associations rather than confirmed causal relationships.

The exclusive occurrence of Genotype 5 in females (*n* = 6) partially correlates, who reported gender-linked HCV distribution patterns in the Middle East. However, given the very small sample size, a definitive conclusion is not statistically feasible [18,21,22].

### 4.3. Temporal Trends and Post-2018 Decline

The significantly younger median age associated with Genotype 3 and 3a has been associated with IDU in international literature [19,21], although risk factor data were not collected in our study. This calls for ongoing age-specific screening and prevention initiatives.

The notable decline in total HCV RNA-positive cases from 2018 onwards (2018: *n* = 244, 2021: *n* = 52) may be attributable to: (a) widespread adoption of pangenotypic DAA therapies, (b) the COVID-19 pandemic reducing healthcare access during 2020–2021, and (c) public health interventions reducing transmission rates.

### 4.4. Clinical and Public Health Implications

Genotype-specific interventions should include: (a) targeted screening for migrant communities with high Genotype 4 prevalence, (b) harm reduction programs for IDU-associated Genotype 3/3a in younger males, (c) culturally appropriate health education materials, and (d) policies ensuring equitable access to pangenotypic DAAs [23,24]. The integration of HCV screening and treatment into fundamental healthcare services for migratory populations is imperative to achieve global HCV elimination objectives [13,25].

### 4.5. Study Limitations

This study has several limitations. First, the single-center, retrospective design limits generalizability. Second, the asylum seeker subgroup was small (*n* = 73, 6.2%), although this proportion reflects the actual migrant ratio in Kayseri (~6%); findings for this group should be considered descriptive. Third, migration status was based solely on nationality records, precluding causal inferences regarding migration and genotype distribution. Fourth, as a laboratory-based study, clinical data—including treatment regimens, DAA response, and patient access pathways—were unavailable. Fifth, two genotyping assays were used sequentially due to platform changes, without a formal concordance study, though both are CE-IVD certified. Sixth, multivariate analysis was not feasible without individual-level raw data. Finally, the marked decline in case numbers after 2018 may partly reflect reduced screening during the COVID-19 pandemic rather than a true epidemiological change. Future multi-center studies with balanced cohorts, clinical integration, and individual-level risk factor data are warranted.

## 5. Conclusions

This eight-year retrospective study (2014–2021) demonstrates that the HCV genotype distribution in Kayseri has undergone significant changes in the context of migration and demographic shifts. While Genotype 1b remains the most prevalent genotype among Turkish citizens, a disproportionately high prevalence of Genotypes 4 and 5 was observed among asylum seekers. The statistically significant increasing trends of Genotype 3 and 3a (*p* < 0.001 and *p* = 0.009, respectively), particularly among younger male populations, suggest evolving transmission dynamics. These findings underscore the necessity of demographically targeted and culturally appropriate screening and treatment strategies for both resident and migrant populations to achieve HCV elimination goals.

## Figures and Tables

**Table 1:** Demographic and Virological Characteristics of HCV RNA-Positive Patients Stratified by Nationality (*n* = 1173), Kayseri, Turkey (2014–2021). Values are presented as *n* (%) unless otherwise stated. * Statistically significant (*p* < 0.05).

Variable	Turkish Nationals *n* (%)	Asylum Seekers *n* (%)	*p*-Value
Total	1100 (93.8)	73 (6.2)	
Gender			
Female	625 (56.8)	43 (58.9)	0.731
Male	475 (43.2)	30 (41.1)	
Age (years)			
Median (range)	66 (14–92)	38 (18–85)	<0.001 *
Genotype			
Genotype 1	33 (3.0)	1 (1.4)	
Subtype 1a	104 (9.5)	17 (23.3)	
Subtype 1b	557 (50.6)	10 (13.7)	
Genotype 2	58 (5.3)	1 (1.4)	
Genotype 3	61 (5.5)	9 (12.3)	
Genotype 3a	18 (1.6)	5 (6.8)	
Genotype 4	268 (24.4)	25 (34.2)	
Genotype 5	1 (0.1)	5 (6.8)	<0.001 *
Year of collection			
2014	54 (4.9)	3 (4.1)	
2015	59 (5.4)	5 (6.8)	
2016	228 (20.7)	17 (23.3)	
2017	240 (21.8)	17 (23.3)	
2018	228 (20.7)	16 (21.9)	
2019	177 (16.1)	10 (13.7)	
2020	64 (5.8)	3 (4.1)	
2021	50 (4.5)	2 (2.7)	0.892

## Data Availability

The data presented in this study are not publicly available due to patient confidentiality but are available from the corresponding author upon reasonable request.
